# Characteristics of rapid eye movement-related obstructive sleep apnea in Thai patients

**DOI:** 10.1038/s41598-022-13382-z

**Published:** 2022-07-05

**Authors:** Nithita Sattaratpaijit, Prapasri Kulalert, Wadee Wongpradit

**Affiliations:** 1 Department of Otolaryngology-Head and Neck Surgery, Faculty of Medicine, Thammasat University, 99/209 Paholyotin Road, Khlong Luang, Pathum Thani Thailand 12120; 2 Department of Clinical Epidemiology, Faculty of Medicine, Thammasat University, 99/209 Paholyotin Road, Khlong Luang, Pathum Thani Thailand 12120; 3 Department of Community Medicine and Family Medicine, Faculty of Medicine, Thammasat University, 99/209 Paholyotin Road, Khlong Luang, Pathum Thani Thailand 12120; 4 Present Address: Center of Excellence in Applied Epidemiology, Thammasat University, 99/209 Paholyotin Road, Khlong Luang, Pathum Thani Thailand 12120

**Keywords:** Health care, Medical research, Risk factors

## Abstract

Obstructive sleep apnea (OSA) is a common sleep disorder that has been associated with cardiovascular consequences. Rapid eye movement (REM)-related obstructive sleep apnea (OSA) is a subtype of OSA which is characterized by apneas or hypopneas predominately during REM sleep. The factors associated with REM-related OSA are still unclear. We aimed to determine the prevalence and associated characteristics of REM-related OSA in Thai patients. A total of 408 patients’ charts were retrospectively reviewed. Demographic and anthropometric characteristics, comorbidities and polysomnographic data were obtained. The patients were divided into two groups: REM-related OSA and non-stage specific OSA. REM-related OSA was defined as an apnea–hypopnea index (AHI) ≥ 5 per hour, with a ratio of REM-AHI to NREM-AHI > 2, and NREM-AHI < 15 per hour. The prevalence of REM-related OSA was 21.6%. AHI and arousal index were both lower in REM-related OSA than in non-stage specific OSA. REM-related OSA was significantly associated with females (OR 2.35, 95% CI 1.25–4.42, *p* = 0.008), age < 60 years (OR 2.52, 95% CI 1.15–5.55, *p* = 0.021), and mild OSA (OR 17.46, 95% CI 9.28–32.84, *p* < 0.001). In conclusion, age < 60 years, female gender, and mild severity of OSA were associated with REM-related OSA.

## Introduction

Obstructive sleep disorder (OSA) is characterized by repetitive narrowing and collapse of the upper airway, resulting in decreased or complete cessation of airflow during sleep^1^, which leads to recurring episodes of hypoxemia, hypercapnia, sleep fragmentation, and increased sympathetic activity^2^. It is a common sleep disorder, affecting between 9 and 38% of adults in the general population^3^.

OSA is a complex heterogeneous disorder so apnea–hypopnea index (AHI) alone cannot discriminate the severity or variety of these conditions^4^. Many categories or phenotypes of patients with OSA have been proposed to classify patients into more homogenous categories. In particular, the distribution of respiratory events between sleep stages of rapid eye movement (REM) sleep and non-rapid eye movement (NREM) sleep are used to categorize OSA patients.

REM-related OSA is characterized by respiratory events such as apnea and hypopnea predominately during REM sleep^5^. The estimated prevalence of REM-related OSA has been reported to be between 10 and 36%^6,7^ of patients with obstructive sleep apnea and it varies according to subjects’ characteristics, and definitions used. In general, the common criteria that is used to identify REM-related OSA is an overall AHI of ≥ 5 and a ratio of the AHI during REM sleep to the AHI during NREM sleep of ≥ 2^5^. Patients with REM-related OSA tend to be younger individuals, women^7,8^ and patients with mild or moderate OSA^6,9^.

REM-related OSA is independently associated with cardiovascular and metabolic complications such as hypertension^10^, metabolic syndrome and diabetes^11^, but the treatment of REM-related OSA is not standardized and patients with this condition may be untreated or undertreated.

Many studies have been published concerning this subtype of OSA, however the factors associated with REM-related OSA remain unclear and no study has reported the prevalence of REM-related OSA in Thai populations. Therefore, the aim of this study was to evaluate the prevalence and to explore characteristics of REM-related OSA in Thailand.

## Material and methods

### Ethics statement

This study was approved by The Human Research Ethics Committee of Thammasat University (Medicine) (IRB project No. 008/2564), in compliance with Declaration of Helsinki, The Belmont Report, CIOMS guidelines and Good Clinical Practice (ICH-GCP) guidelines. All methods were performed in accordance with these guidelines and regulations. All participants provided written informed consent.

### Study design and subjects

**Figure 1:**
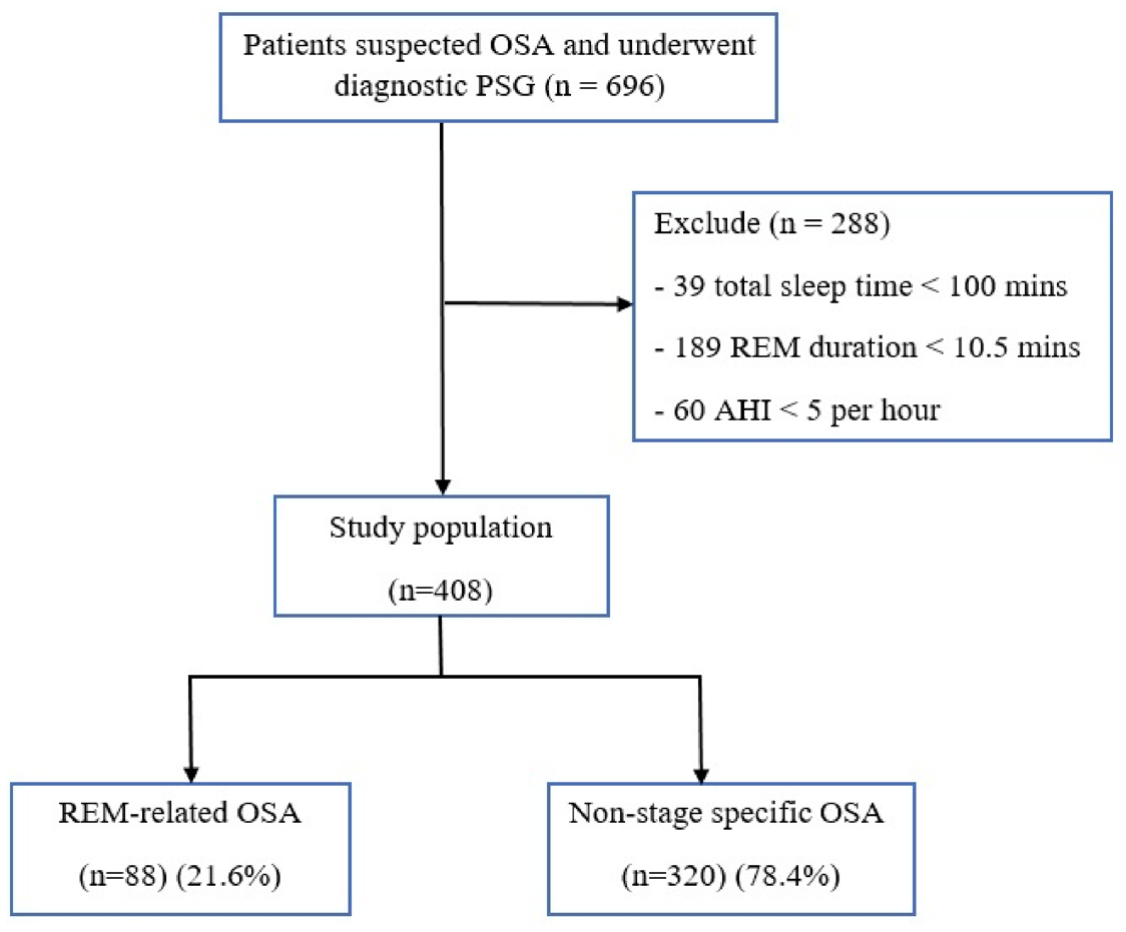
Study population flow chart. *OSA* obstructive sleep apnea, *PSG* polysomnography, *AHI* apnea–hypopnea index, *REM* rapid eye movement.

This was retrospective chart review study. We included all patients who were over 18 years old with suspected OSA and underwent standard diagnostic polysomnography (either split-night or full-night) between November 2019 and October 2020. The study was conducted at sleep lab, Thammasat University Hospital, Thammasat University, Pathum Thani, Thailand. The suspected OSA patients were assessed by symptoms suggesting OSA, such as snoring, observed apnea, nocturnal choking, nocturia, and daytime sleepiness from the request document for PSG. Patients were excluded from the study if AHI < 5 per hour, total sleep time < 100 min, REM sleep duration < 10.5 min, or polysomnographic data was incomplete. Figure 1 shows the study population flow chart.

Demographic data (age, sex), self-reported questionnaire, Thai Epworth sleepiness scale and anthropometric measurement were routinely recorded prior to PSG.

The comorbidities were obtained from medical records or current drug treatment.

Anthropometric measurements were taken by trained sleep technicians using standard techniques^12^. Weight in kilograms (kg) was measured while wearing light clothing and without shoes, using a digital scale to the nearest 100 g. Height in meters (m) was measured without shoes, with stadiometer to the nearest 0.5 cm (cm). Body mass index (BMI) was calculated as weight (kg) divided by the square of height (m^2^). The patients were categorized into 2 groups according to the Asian-Pacific cutoff value^13^ for obesity, which was ≥ 25 kg/m^2^. Neck circumference (NC) was measured (cm) using an inelastic tape with 1 mm precision at the level of the cricothyroid membrane in upright position^14^. The cutoff point 40 cm was used for large NC.

Daytime sleepiness was evaluated using the Thai version of the Epworth Sleepiness Scale (ESS) questionnaires^15^. A score > 10 was considered to indicate excessive daytime sleepiness.

### Polysomnography and scoring

A standard polysomnography (PSG) was performed according to American Academy of Sleep Medicine (AASM) recommendations^16^ by using a digital polysomnographic monitor (Greal Series, Compumedics, Victory, Australia). The following biological variables were monitored continuously: electroencephalography (F4-M1, C4-M1, O2-M1, F3-M2, C3-M2 and O1-M2), electrooculography, submental and anterior tibial electromyography, and electrocardiography. Inductance plethysmography belts were used to monitor chest and abdominal movements. Thermistor and nasal pressure transducers were used to monitor airflow. Snoring was recorded using a microphone. Pulse oximetry was used to measure arterial oxygen saturation (SpO2). The cumulative percentage of time spent at saturation below 90% was calculated from pulse oximetry. Data were scored manually by a sleep specialist according to the AASM scoring system^16^.

Apnea was defined as a ≥ 90% reduction of airflow with a duration of at least 10 s, while hypopnea was defined as a ≥ 30% reduction of airflow that was associated with either arousal or with a ≥ 3% arterial oxygen desaturation, for a duration of at least 10 s.

AHI was calculated as the mean number of events of either apnea or hypopnea per hour of total sleep time. REM-AHI was the mean number of events, either apnea or hypopnea, per hour of REM sleep. NREM-AHI was the mean number of events, either apnea or hypopnea, per hour of NREM sleep.

### Definitions

The diagnosis of OSA was made by evidence of AHI ≥ 5. The OSA severity was classified as: mild for 5 ≤ AHI < 15, moderate for 15 ≤ AHI < 30, and severe for AHI ≥ 30^17^.

Patients were considered to have REM-related OSA if the ratio of REM-AHI to NREM-AHI was > 2 and NREM-AHI < 15^8^. Patients who did not fulfill the definition of REM-related OSA were considered to have non-stage specific OSA. After sorting the patients into these two groups, the demographic and anthropometric characteristics, comorbidities, and polysomnographic characteristics of the REM-related OSA group and the non-stage specific OSA group were compared.

### Statistical analysis

The data was analyzed using Stata version 14 (STATA Corp., Texas, USA). The prevalence is reported as percentage of population. Continuous variables are presented as mean ± standard deviation for normally distributed variables or median and interquartile range (IQR) for non-normally distributed variables. Categorical variables are presented as number and percentage. Comparisons between groups were analyzed using the unpaired t test for continuous variables with normal distribution, the Mann–Whitney U test for continuous variables with non-normal distribution, and the Chi-square test for categorical variables.

Potential associated factors of REM-related OSA were analyzed using multivariate logistic regression analysis to calculate the odds ratios (ORs) and 95% confidence intervals (CIs). Demographic and clinical variables including age, gender, NC, BMI, excessive daytime sleepiness, hypertension, asthma, diabetes and OSA severity were examined in the univariate analysis. All variables on univariate analysis were also used for analysis in the multivariate logistic regression model. All statistical tests were two-sided, and *p*-value < 0.05 was considered to be statistically significant.

## Results

### Prevalence of REM-related OSA

Of the 696 patients suspected of OSA who underwent diagnostic PSG, 288 patients were excluded from study and 408 patients were enrolled and diagnosed as OSA (Fig. 1). The participants consisted of 245 (60.1%) men and 163 (39.9%) women; their median age was 49.7 ± 15.8 years.

The prevalence of REM-related OSA was 21.6%. REM-related OSA was more prevalent in younger patients (45.2 ± 14.9 vs. 50.9 ± 15.8, *p* = 0.002) and in women (54.5% vs. 35.9%, *p* = 0.002).

### Comparison of demographic, anthropometric, ESS, comorbidities characteristics between patients with REM-related OSA and patients with non-stage specific OSA

The REM-related OSA group of patients tended to have thinner necks than the non-stage specific OSA group; (75% vs. 56.3%, *p* = 0.001) had neck circumferences ≤ 40 cm. The non-stage specific OSA group had a higher proportion of comorbid diabetes (25.9% vs. 13.6%, *p* = 0.016). There were no significant differences in BMI, Epworth sleepiness scale, or excessive daytime sleepiness between the two groups, as shown in Table 1.

**Table 1:** Demographic, anthropometric, ESS, comorbidities characteristics of patients with REM-related OSA and non-stage specific OSA.

Characteristics	Total (n = 408)	REM-related OSA (n = 88)	Non-stage specific OSA (n = 320)	*p*-value
**Age, years**	49.7 ± 15.8	45.2 ± 14.9	50.9 ± 15.8	0.002
**Age**				0.004
< 60 years	288 (70.6)	73 (82.9)	215 (67.2)	
≥ 60 years	120 (29.4)	15 (17.1)	105 (32.8)	
**Gender**				0.002
Male	245 (60.1)	40 (45.5)	205 (64.1)	
Female	163 (39.9)	48 (54.5)	115 (35.9)	
**Neck circumferences**				0.001
≤ 40 cm	246 (60.3)	66 (75.0)	180 (56.3)	
> 40 cm	162 (39.7)	22 (25.0)	140 (43.7)	
**BMI classification**				0.239
< 25 kg/m^2^	88 (21.6)	23 (26.1)	65 (20.3)	
≥ 25 kg/m^2^	320 (78.4)	65 (73.9)	255 (79.7)	
**ESS**	8.7 ± 4.9	8.4 ± 5.3	8.8 ± 4.8	0.462
**Excessive daytime sleepiness**				0.338
No	138 (33.8)	26 (29.5)	112 (35.0)	
Yes	270 (66.2)	62 (70.5)	208 (65.0)	
**Comorbidities**				
Hypertension	208 (51.0)	44 (50.0)	164 (51.2)	0.835
Diabetes mellitus	95 (23.3)	12 (13.6)	83 (25.9)	0.016
Asthma	12 (2.9)	1 (1.1)	11 (3.4)	0.475
COPD	2 (0.5)	0 (0.0)	2 (0.6)	> 0.999
Cardiovascular disease	64 (15.7)	9 (10.2)	55 (17.2)	0.112
Pulmonary hypertension	15 (3.7)	1 (1.1)	14 (4.4)	0.209

Data are expressed as mean ± SD, or n (%).

*REM* rapid eye movement, *OSA* obstructive sleep apnea, *BMI* body mass index, *ESS* Epworth Sleepiness Scale.

### Comparison of polysomnographic characteristics between patients with REM-related OSA and patients with non-stage specific OSA

Patients with REM-related OSA showed higher sleep efficiency, percentage of stage N3, mean SpO2 and minimum SpO2 than patients with non-stage specific OSA. They also showed lower percentage of stage N1, arousal index, and duration of SpO2 < 90% (Table 2).

**Table 2:** Polysomnographic features of patients with REM-related OSA and non-stage specific OSA.

Variables	Total (n = 408)	REM-related OSA (n = 88)	Non-stage specific OSA (n = 320)	*p*-value
Sleep efficiency (%)	78.9 (66.2, 87.0)	83.7 (73.1, 89.9)	77.9 (63.9, 86.2)	< 0.001
Stage N1 (%)	16.0 (11.0, 25.0)	11.8 (6.7, 14.0)	18.0 (12.0, 27.0)	< 0.001
Stage N2 (%)	49.6 ± 11.7	51.4 ± 11.8	49.1 ± 11.2	0.110
Stage N3 (%)	16.0 (6.7, 25.0)	20.3 (11.5, 29.7)	15.3 (5.8, 23.9)	< 0.001
Stage REM duration (min)	27.5 (17.0, 48.5)	43 (22.0, 70.5)	26 (17.0, 41.5)	< 0.001
Stage REM (%)	14.3 ± 5.6	15.4 ± 5.8	14.0 ± 5.5	0.047
AHI (events/h)	29.2 (14.7, 54.0)	11.3 (7.9, 15.7)	39.9 (22.7, 63.5)	< 0.001
**PSG severity**				< 0.001
Mild	103 (25.3)	62 (70.4)	41 (12.8)	
Moderate	107 (26.2)	26 (29.6)	81 (25.3)	
Severe	198 (48.5)	0 (0.0)	198 (61.9)	
REM-AHI (events/h)	45.4 (22.6, 65.1)	37.6 (24.7, 54.6)	49.1 (19.9, 67.9)	0.029
NREM-AHI (events/h)	27.3 (12.0, 53.4)	7.1 (4.4, 10.8)	36.7 (20.7, 61.9)	< 0.001
Apnea index (events/h)	2.1 (0.2, 9.9)	0.3 (0, 1.6)	3.5 (0.4, 16.7)	< 0.001
Hypopnea index (events/h)	20.3 (10.5, 35.6)	9.9 (6.8, 13.7)	26.1 (16.1, 39.7)	< 0.001
Arousal index (events/h)	24.6 (13.9, 40.5)	10.5 (5.8, 14.9)	29.5 (18.1, 47.0)	< 0.001
Mean SpO2 (%)	95.0 (92.0, 96.0)	96.0 (94.0, 97.0)	94.0 (91.0, 96.0)	< 0.001
Lowest SpO2 (%)	83.0 (72.5, 89.0)	86.0 (79.0, 89.0)	82.5 (69.0, 89.0)	0.002
SpO2 time < 90% (min)	3.0 (0.3, 12.6)	0.9 (0.2, 3.8)	4.3 (0.4, 18.0)	< 0.001

Data are expressed as mean ± SD, median (IQR), or n (%).

*REM* rapid eye movement, *NREM* non-rapid eye movement, *OSA* obstructive sleep apnea, *AHI* apnea–hypopnea index, *SpO2* arterial oxygen saturation.

AHI was significantly lower in patients with REM-related OSA compared with non-stage specific OSA patients (11.3 (7.9, 15.7) vs. 39.9 (22.7, 63.5), *p* < 0.001). In REM-related OSA patients, mild OSA and moderate OSA were reported in 70.4% and 29.6% of patients, while no severe OSA events were reported.

### Regression analysis of factors associated with REM-related OSA

The results of logistic regression analysis are shown as Table 3. According to the univariate analysis, the factors associated with REM-related OSA were female gender, age ≤ 60 years, neck circumference ≤ 40 cm, mild OSA, and diabetes. After multivariate analysis, REM-related OSA was significantly statistically associated with female gender (aOR 2.35, 95% CI 1.25–4.42, *p* = 0.008), age < 60 years (aOR 2.52, 95% 1.15–5.55, *p* = 0.021), and mild OSA (aOR 17.46, 95% CI 9.28–32.84, *p* < 0.001). Therefore, asthma and diabetes had negative association with REM-related OSA.

**Table 3:** Regression analysis of factors associated with REM-related OSA.

Variables	Univariate	*p*-value	Multivariate	*p*-value
	OR (95% CI)		aOR* (95% CI)	
Age < 60 years	2.38 (1.30–4.34)	0.005	2.52 (1.15–5.55)	0.021
Female	2.14 (1.33–3.45)	0.002	2.35 (1.25–4.42)	0.008
NC ≤ 40 cm	2.33 (1.37–3.97)	0.002	1.32 (0.65–2.71)	0.443
BMI ≥ 25 kg/m^2^	0.72 (0.42–1.25)	0.241	1.33 (0.62–2.85)	0.457
No excessive daytime sleepiness (ESS ≤ 10)	1.28 (0.77–2.14)	0.339	1.14 (0.60–2.17)	0.680
Hypertension	0.95 (0.59–1.52)	0.835	1.59 (0.82–3.09)	0.169
Asthma	0.32 (0.04–2.54)	0.282	0.10 (0.01–0.95)	0.045
Diabetes	0.45 (0.23–0.87)	0.018	0.39 (0.16–0.95)	0.039
**OSA severity**				
Mild	16.23 (9.24–23.50)	< 0.001	17.46 (9.28–32.84)	< 0.001
Moderate to severe	Reference		Reference	

*NC* neck circumferences, *BMI* body mass index, *ESS* Epworth Sleepiness scale.

*Adjusted odds ratio obtained from multivariate logistic regression adjusting for all variables presented in this table.

## Discussion

This present study was designed to determine the prevalence and associated factors including demographic and polysomnographic features of REM-related OSA in Thai patients. The results of this study showed that the prevalence of REM-related OSA was 21.6%. REM-related OSA was more prevalent in younger patients and in women.

Previous studies have considered the prevalence of REM-related OSA, but estimates vary from between 10 and 36%^6,7^ of OSA patients, partly because definitions of REM-related OSA differ. The most common criterion for REM-related OSA is a ratio of AHI during REM sleep to AHI during NREM sleep of ≥ 2^5^. However, this definition may misdiagnose patients who are significantly affected by OSA during NREM if the ratio of REM-AHI to NREM-AHI is greater than 2. Therefore, we added the condition that NREM-AHI < 15. This approach has also proven clinically useful in some previous studies^8,18^. The prevalence of REM-related OSA in this study was equivalent to that in previous studies^5,18^ which used the same definition.

In this study, comparing REM-related OSA with non-stage specific OSA showed that the patients with REM-related OSA were younger individuals and more often female. Likewise, the logistic regression confirmed that age < 60 years and female gender were independent correlates of REM-related OSA. These results are consistent with other studies^5,7,19^.

However, the pathophysiology has not been well clarified. A possible explanation may relate to gender differences in obstructive sleep apnea such that the obstructive events are predominant in men in NREM and in women in REM. Compared to men, women have different upper airway anatomy, non-anatomical factors and sex hormones. Impaired upper airway anatomy is the key cause of OSA. The upper airway is stiffer in women than in men, whereas the pharyngeal airway is longer and there is more soft tissue thickening on the lateral pharyngeal wall in men than women, so women are less susceptible to airway collapse^20^.

The results of a large cohort study suggest that gender differences in OSA are influenced by state-specific mechanisms. Women demonstrated lower loop gain and lower arousal threshold in NREM^21^. In addition, female hormones (progesterone) may increase the upper airway dilator muscle tone and stimulate ventilation by increasing the chemoreceptor response to hypoxia and hypertonia^22^. But protective mechanisms in NREM do not substantively protect women from airway collapse during REM sleep which is characterized by muscle atonia and decreased chemosensitivity^23^.

REM-related OSA patients tend to have lower overall AHI than non-stage specific OSA patients. In the REM-related group, 70.4% of patients had mild OSA, 29.6% had moderate OSA, and none had severe OSA. In the non-stage specific group, 61.9% had severe OSA. REM sleep accounts for approximately a quarter of total sleep duration^2^, so the overall AHI predominantly reflects NREM-AHI. Additionally, the polysomnographic findings showed that sleep quality was better and arousal index was lower in REM-related OSA than in non-stage specific OSA, which is more severe. Increased BMI has been reported to increase severity of OSA^24^, but our study found no significance differences related to BMI.

Excessive daytime sleepiness is considered an important symptom of OSA. In previous studies, the association of excessive daytime sleepiness and REM-related OSA was equivocal. The current study showed no significant differences of excessive daytime sleepiness between patients with REM-related OSA and those with non-stage specific OSA, based on Epworth Sleepiness Scale, despite lower overall AHI in REM-related OSA. Interruption of REM sleep may have more impact on daytime sleepiness than interruption of NREM sleep^25^. Therefore, although REM-AHI in non-stage specific OSA was significantly higher, both groups showed severe REM-AHI (REM-AHI ≥ 30 per hour) which could reflect similar symptoms. Oxygen desaturation during REM sleep might cause excessive daytime sleepiness^19^, but our data did not show any difference.

Two population-based studies demonstrated significant and clinically relevant associations between REM-AHI and prevalent hypertension. NREM-AHI was not associated with hypertension after adjusting for REM-AHI^10,26^. The HypnoLaus Sleep Cohort study verified that REM-AHI ≥ 20 per hour was associated with increased risk of hypertension^27^. The strong association of hypertension with adverse cardiovascular outcomes link REM-related OSA to cardiovascular outcomes^28^. Interestingly, our data showed REM-AHI ≥ 20 per hour in both groups, so the potential of hypertension and cardiovascular disease was similar in both REM-related OSA and non-stage specific OSA.

In contrast, diabetes and asthma showed negative association with REM-related OSA. However, the results must be interpreted with caution because our study reported that diabetes and asthma were more common in non-stage specific OSA.

Plausible pathophysiological and causal links between OSA and glucose metabolism dysregulation are sleep fragmentation and intermittent hypoxemia^29^. In our study, non-stage specific OSA showed higher overall AHI, REM-AHI, arousal index (which represented sleep fragmentation) and oxygen desaturation.

The Sleep Heart Heath Study demonstrated that increased REM-AHI was independently associated with insulin resistance after adjustment for multiple potential confounders^11^ and suggested that REM-related OSA is likely to have an impact on glucose metabolism and diabetes, even absence of NREM-AHI. In addition, the relation between REM-related OSA and diabetes relied on the severity of REM-AHI. The HypnoLaus Sleep Cohort study showed REM-AHI ≥ 20 per hour or increasing REM-AHI severity were significantly associated with diabetes in both subgroups with NREM-AHI < 10 per hour and AHI < 10 per hour^27^.

The association between REM-related OSA and asthma was interesting because a prospective observational study showed that asthma was independently associated with REM-related OSA^19^, which was consistent with a previous study in children with OSA^31^, while our results showed a negative relationship. However, the published pathophysiologic mechanisms that explain the association between asthma and OSA, especially REM-related OSA, are not well understood and are limited. Asthma and OSA are heterogeneous conditions which exhibit complex bidirectional interactions, shared risk factors and co-morbidities between each other^32^.

There may be some possible limitations in this study. Firstly, in our routine practice, split-night PSG was applied more frequently than full-night PSG because it costs less.

Therefore, the duration of the REM sleep, which is mostly concentrated in the second half of the sleep period, was lower in split-night PSG than in full-night PSG^33^. In fact, the standard diagnostic criteria did not clarify minimum duration for REM sleep. To reduce this bias, we selected 10.5 min as the minimum REM sleep duration, but a longer REM duration may diagnose REM-related OSA more precisely. Secondly, we used moderate and severe OSA as reference in regression analysis because there was no severe OSA in the REM-related group. Our result showed mild severity of OSA was the independent associated factor. However, moderate severity was also found in REM-related OSA.

The contribution of this study is to confirm that REM-related OSA is highly prevalent in Thai OSA patients and has clinical implications related to sleepiness and comorbidities. Further studies are required to better understand the roles of asthma and diabetes in REM-related OSA. Moreover, impacts and options of therapy should be investigated.

In summary, this study has determined that age < 60 years, female gender, and mild severity of OSA were associated with REM-related OSA.
